# Wie die Lichtwahrnehmung unsere innere Uhr beeinflusst: (How photoreception affects our inner clock)

**Published:** 2023-07

**Authors:** Stephan Munkwitz, Manuel Spitschan

**Affiliations:** 1 Max-Planck-Institut für biologische Kybernetik, Tübingen; 2 Augenärzte Mittlerer Neckar, Praxis Stuttgart-Mitte, Stuttgart; 3 Technische Universität München, Fakultät für Sport- und Gesundheitswissenschaften, München; 4 Technische Universität München, Institute for Advanced Study, Garching

## Abstract

Neben den Stäbchen und Zapfen sind die melanopsinhaltigen intrinsischen photosensitiven Ganglienzellen (ipRGC) der dritte Typ von Photorezeptoren in der Netzhaut. Die Hauptaufgabe der ipRGC ist es, dem Gehirn Informationen zu den Lichtverhältnissen zu signalisieren. Über die retinohypothalamische Nervenbahn synchronisiert Licht unsere innere Uhr und passt sich so dem Licht-Dunkel-Wechsel unserer Umgebung an. Das Licht ist für unsere innere Uhr dabei der wichtigste Zeitgeber und die ipRGC dadurch von zentraler Bedeutung für die Chronobiologie. Darüber hinaus folgen viele okuläre und retinale Stoffwechselvorgänge einem zirkadianen Rhythmus. Die Chrono-Ophthalmologie ist ein neu entstehendes Forschungsgebiet, das die Schnittstelle zwischen Auge und zirkadianem Rhythmus untersucht.

## Summary

In addition to the rods and cones, the melanopsin-containing intrinsically photosensitive retinal ganglion cells (ipRGC) are responsible for encoding ambient illumination conditions. The main task of the ipRGC is to signal information about illumination to the brain. Via the retinohypothalamic pathway, light synchronises our internal biological clock and sets it to follow the environment’s light-dark cycle. Light is the most important zeitgeber for our inner clock and the ipRGC are therefore highly important for the field of chronobiology. Many ocular and retinal functions also follow a diurnal rhythm. Chrono-ophthalmology is an emerging research field at the interface between the eye and the clock.

Durch die Rotation der Erde um ihre eigene Achse entstehen tagesperiodische Veränderungen der Umwelt: Es herrschen Tag und Nacht, es existieren wiederkehrende Jahreszeiten. Die Umweltfaktoren unterliegen einem rhythmischen Auf und Ab. Im Laufe der Evolution haben sich das Tierund Pflanzenreich an diese rhythmischen Veränderungen angepasst.

Viele Arten haben eine „innere Uhr” entwickelt und sich sozusagen mit ihrer Umwelt synchronisiert. Nahezu alle Lebewesen, vom Einzeller bis zum Menschen, weisen rhythmische Zustandsänderungen ihrer Organe und Funktionen auf [24, 25].

## Zirkadianer Rhythmus

Viele Stoffwechselvorgänge, die Körpertemperatur, der Muskeltonus oder die Konzentrationsfähigkeit, um nur einige zu nennen, folgen einem zirkadianen Rhythmus. Am auffälligsten ist beim Menschen der Schlaf-Wach-Rhythmus.

Da die Periode des endogenen Rhythmus näherungsweise 24 Stunden beträgt, wurde der Begriff “zirkadian” (von lat. circa dies = ungefähr ein Tag) gewählt. Bei den meisten Menschen ist eine zirkadiane Periode etwas länger als 24 Stunden und beträgt bei Sehenden im Mittel 24,18 Stunden, bei Frauen (24,05 Stunden ± 12 min) ist sie signifikant kürzer als bei Männern (24,11 Stunden ± 12 min) [6, 8].

Unsere innere Uhr wird durch eine zentrale zirkadiane Uhr mit dem Licht-Dunkel-Wechsel synchronisiert und hat dadurch einen 24-Stunden-Rhythmus. Ihre wichtigste Funktion ist es, den Schlaf-Wach-Rhythmus zu steuern.

Interessant ist, dass die zirkadianen Rhythmen auch bei völliger Isolation von den äußeren Zeitgebern über Monate stabil bleiben können: Nur durch die “innere Uhr” gesteuert, ohne die Synchronisation durch äußere Zeitgeber laufen sie lediglich etwas langsamer oder etwas schneller als normal.

## Zentrale zirkadiane Uhr liegt im Nucleus suprachiasmaticus

**Abbildung 1:**
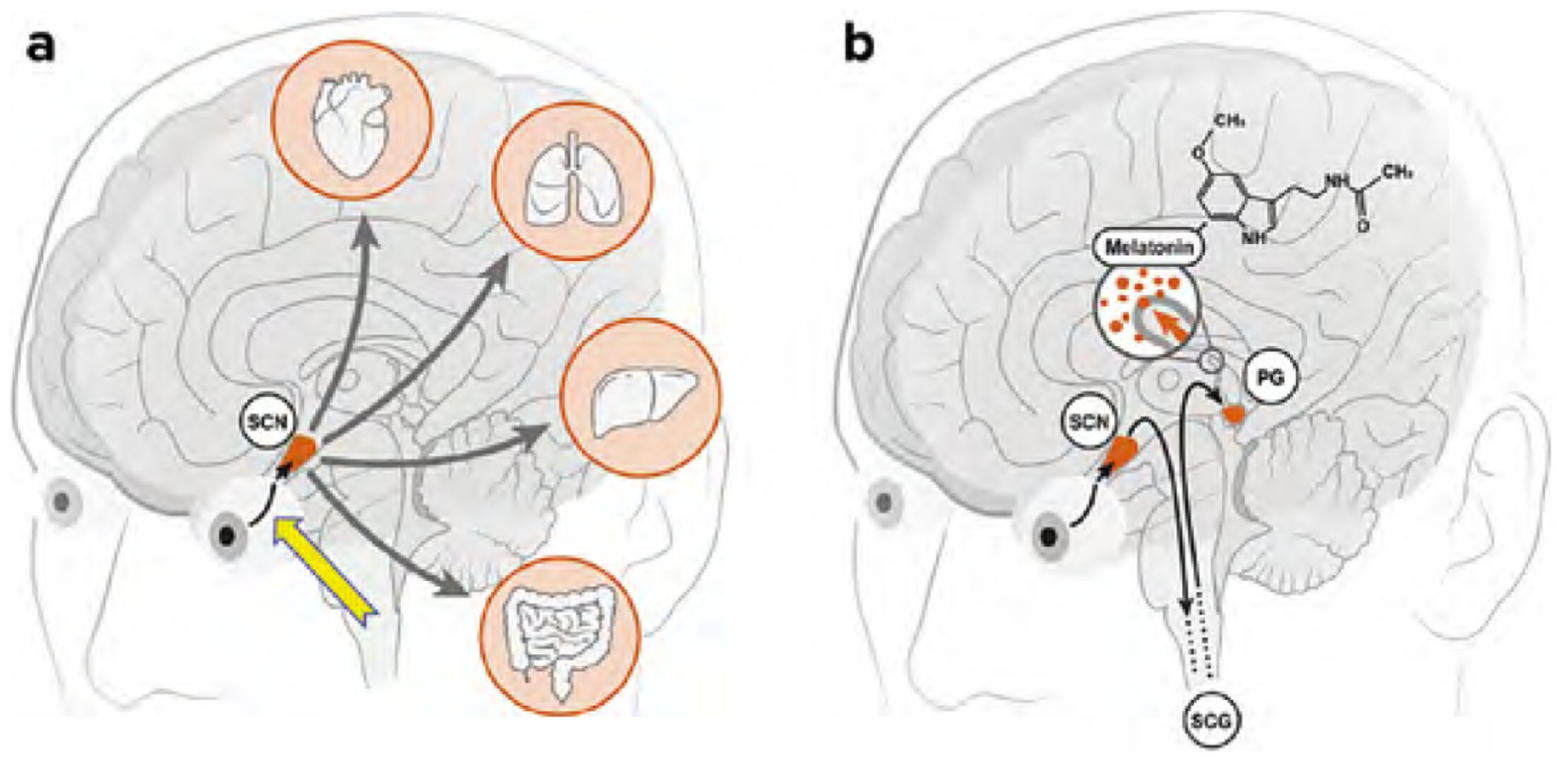
a) Die retinohypothalamische Nervenbahn (gelber Pfeil) verbindet die Netzhaut, speziell die melanopsinhaltigen photosensitiven Ganglienzellen mit dem Nucleus suprachiasmaticus (SCN). Die Nervenbahn tritt aus dem Nervus opticus aus und innerviert den Bereich im anterioren Hypothalamus. Dieser liegt über dem Chiasma opticum. Der dort befindliche Nucleus suprachiasmaticus wird getriggert und sorgt so für die Erhaltung des zirkadianen Rhythmus. Die ausgehenden Signale des SCN wirken auf die peripheren Schrittmacher in Herz, Lunge, Leber und Gastrointestinaltrakt. b) Durch Licht wird über diese retinohypothalamische Nervenbahn die Produktion des körpereigenen “Schlafhormons” Melatonin unterdrückt. Hierbei werden die Signale vom SCN über den Hirnstamm zum Rückenmark, weiter bis zum oberen Halsganglion (SCG) und dann zurück ins Gehirn zur Zirbeldrüse (PG) weitergeleitet. Die vom oberen Halsganglion kommenden Fasern schütten an ihren Endigungen Noradrenalin aus. Der Überträgerstoff besetzt spezielle molekulare Rezeptoren auf der Zellmembran der Pinealzellen und hemmt dadurch die Melatonin-Synthese. (PG, pineal gland = Zirbeldrüse; SCG, superior cervical ganglion = oberes Halsganglion).

Die zentrale zirkadiane Uhr befindet sich im zentralen Nervensystem (ZNS) und liegt im Nucleus suprachiasmaticus (”suprachiasmatic nucleus”, SCN) im ventralen Hypothalamus. Er ist das nervliche Substrat der inneren Uhr. Er erhält sensorische Informationen von der Retina und steuert zirkadiane Rhythmen des vegetativen Nervensystems. Er wirkt als Taktgeber auf sehr viele physiologische Prozesse wie das Immunsystem, den Schlafrhythmus, die Körpertemperatur, oder den Hormonhaushalt und steuert damit auch unsere Leistungsfähigkeit, den Appetit und andere Vorgänge. Der SCN hat Verbindungen zu peripheren Schrittmachern z. B. in Herz, Lunge, Leber und Gastrointestinaltrakt (Abbildung 1 a). Die Nervenzellen im SCN oszillieren auch in Abwesenheit von externen Reizen innerhalb einer Periode von rund 24 Stunden [12].

Die innere Uhr reagiert auf sogenannte Zeitgeber. Dies sind Reize aus der Umgebung und auch körperinterne Signale, die helfen, unsere zentrale zirkadiane Uhr mit unserer Umgebung zu synchronisieren. Licht ist hierbei der wichtigste Zeitgeber.

## Intrinsische lichtempfindliche Ganglienzellen

Es ist bekannt, dass neben den Zapfen und Stäbchen die intrinsischen lichtempfindlichen Ganglienzellen (”intrinsically photosensitive retinal ganglion cells”, ipRGC) einen weiteren lichtempfindlichen Mechanismus im Auge darstellen, welcher unabhängig von den Zapfen und Stäbchen abläuft [7, 11, 14, 19, 22, 26]. Dieser Mechanismus wird auch als “nicht visuelle” Wirkung des Lichtes bezeichnet und wird durch das Photopigment Melanopsin gesteuert. Die ipRGC reagieren auf melanopisch wirksame Lichtanteile, besonders auf blaues Licht, da die spektrale Empfindlichkeit von Melanopsin bei 480 nm und damit im kurzwelligen Bereich liegt.

Intrinsisch lichtempfindliche Ganglienzellen machen 1–2 % der retinalen Ganglienzellen aus. Sie sind über die ganze Netzhaut verteilt. Im Unterschied zu den anderen Ganglienzellen exprimieren nur die ipRGC das lichtempfindliche Protein Melanopsin. Die ipRGC liefern nicht die Information zur Bild-, Muster-oder Farberkennung, sondern erfassen die Umgebungshelligkeit. Mit diesen Informationen können die Körperfunktionen an die Lichtverhältnisse der Umgebung angeglichen werden [15]. Die ipRGC leiten die Signale der Netzhaut über den retinohypothalamischen Trakt an den SCN im Zwischenhirn weiter (Abbildung 1 b). Hier führt das Lichtsignal über diesen retinohypothalamischen Nerventrakt zur Unterdrückung der Melatoninproduktion mit zirkadianer Synchronisierung und auch zu einer Verbesserung der subjektiven Aufmerksamkeit, welche auch mittels EEG- und MRT-Untersuchungen bestätigt wurde [3].

## Einfluss der ipRGC auf die Stimmung

Es gehört zur menschlichen Grunderfahrung, dass Licht die Stimmung beeinflusst. Dies wird beispielsweise in Form der Lichttherapie bei der Behandlung depressiver Stimmungslagen genutzt und wirkt sich insbesondere in den dunkleren Jahreszeiten therapeutisch positiv aus. Es ist auch bekannt, dass dabei ipRGC eine zentrale Rolle spielen [9].

Jüngst konnten Fernandez und Mitarbeiter in experimentellen Studien mit Mäusen nachweisen, dass die ipRGC mit einer bisher unerkannten Region im Thalamus, dem Nucleus perihabenularis (PHb) kommunizieren, unabhängig von der Schrittmacherfunktion des SCN. Der PHb ist in einen besonderen Schaltkreis mit die Stimmung regulierenden Zentren integriert. Er hat neuronale Verbindungen zum präfrontalen Kortex, der elementar wichtig ist für die Stimmungs-regulation. Die ipRGC steuern so die Auswirkungen von Licht auf das affektive Verhalten.

## Einfluss auf die innere Uhr

### Netzhauterkrankungen

Netzhauterkrankungen wie hereditäre Netzhautdystrophien (z. B. Retinitis pigmentosa, Zapfen-Stäbchendystrophien, Makuladystrophien) oder Netzhautdegenerationen (z. B. altersbedingte Makuladegeneration oder myope Makulopathie) führen je nach Stadium zu einem unterschiedlichen Rückgang der Netzhautschichten und einem damit verbundenem Rückgang der Stäbchen, Zapfen und auch der ipRGC. Es ist denkbar, dass der Rückgang der ipRGC auch einen Einfluss auf die innere Uhr der Patienten hat. Hierzu sind noch viele Fragen in der Forschung offen.

### Erkrankungen des Optikus

Auch bei Erkrankungen des Optikus konnten verschiedene Auswirkungen auf die Funktion der ipRGC nachgewiesen werden. So ist beispielsweise die Prävalenz von Schlafund zirkadianen Störungen bei Glaukompatienten hoch.

Auch konnte bei Patienten mit einseitiger oder beidseitiger anteriorer ischämischer Optikusneuropathie (AION) eine funktionelle Schädigung der ipRGC-vermittelten Signale im Vergleich zu den nicht betroffenen Augen und den Kontrollaugen festgestellt werden [17].

Auf der anderen Seite scheint ein unbekannter Schutzmechanismus die ipRGC bei mitochondrialen Optikusneuropathien wie bei der Leberschen hereditären Optikusneuropathie (LHON) oder der dominanten Optikusatrophie (DOA) vor dem Untergang zu bewahren: Histologische Studien belegen, dass bei diesen Erkrankungen die mRGC im Vergleich zum massiven Rückgang normaler RGC sowohl bei LHON als auch bei DOA relativ gut erhalten sind, was den intakten Pupillenreflex bei diesen Patienten erklärt [17].

## Nicht-24-Stunden-Schlaf-Wach-Störung

Die Nicht-24-Stunden-Schlaf-Wach-Störung (”Non-24-Hour Sleep-Wake Disorder”, N24SWD), kurz Non-24, ist eine sehr seltene Störung des Schlaf-Wach-Rhythmus. Bis zu 70 % der vollständig erblindeten Personen sind jedoch davon betroffen, weil die Lichtempfindung als Zeitgeber für die Adjustierung des zirkadianen Schrittmachers fehlt und ihr intrinsischer zirkadianer Rhythmus nicht mehr mit dem 24-Stunden-Tag der Umgebung synchronisiert ist [10].

Aber ein Teil der Blinden und Sehbehinderten weist zirkadiane Rhythmen auf [1]. Die Funktionalität der ipRGC und der Nervenfasern zum SCN ist hier von entscheidender Bedeutung. Im Einzelnen muss analysiert werden, welche Schädigung vorliegt. Bei intakter innerer Netzhaut und guter Funktionalität der ipRGC können Blinde und Sehbehinderte den Melatoninhaushalt weiterhin steuern [5, 13, 29].

### Schlafanamnese sinnvoll

Für die Klinik und Praxis bedeutet dies, dass eine kurze Schlafanamnese bei jedem Blinden und Sehbehinderten standardmäßig dazugehören sollte, um eine Schlafstörung ausschließen zu können. Ohne apparativen Aufwand könnten so alle Ärzte und nicht nur Schlafexperten zum Wohl dieser Patientengruppe und auch zur verbesserten Klassifizierung der unterschiedlichen Augenerkrankungen beitragen.

Darüber hinaus kann ein Wissen über die ipRGC und ihre Bedeutung für den Schlaf-Wach-Rhythmus auch Blinde und Sehbehinderte für Schlafschwierigkeiten sensibilisieren und ihnen dadurch helfen positiver mit ihrer Behinderung umzugehen.

## Chrono-Ophthalmologie

Die Forschungsgruppe “Translational Sensory and Circadian Neuroscience” des Max-Planck-Instituts für biologische Kybernetik unter Leitung von Prof. Dr. Manuel Spitschan interessiert sich für die Chronobiologie und ihre Mechanismen der zirkadianen und neuroendokrinen Lichtwahrnehmung des Menschen. Die Chronobiologie untersucht als Wissenschaftszweig der Biologie die zeitliche Organisation von physiologischen Prozessen und wiederholten Verhaltensmustern bei Organismen [23].

Die Chrono-Ophthalmologie verbindet die Chronobiologie mit der Ophthalmologie und hat zum Ziel, die zirkadianen Rhythmen in Auge und Netzhaut zu erkennen und darzustellen. Die neuen Erkenntnisse könnten insbesondere für die zeitliche Steuerung der Therapie – wann gebe ich welchen Wirkstoff – oder die optimale individuelle Ausnutzung der besten Tages-oder Nachtsehschärfe wegweisend sein.

Neben den geschilderten Auswirkungen der ipRGCs sind weitere tageszeitabhängige oder zirkadiane Phänomene bekannt: So ist eine zirkadiane Veränderung der Pupillenantwort und der Pupillengröße belegt [21, 27, 30]. Auch tageszeitabhängige Schwankungen des Augeninnendrucks sind weitreichend bekannt und haben schon lange mit den ambulanten Tagesdruck-profilen sowie stationären Tagesdruck-profilen mit Nachtmessung Einzug in die klinische Routine gefunden [16, 20].

Veränderungen der Augenoberfläche im Tagesverlauf sind im Zusammenhang mit dem trockenen Auge bekannt. Tageszeitliche Visusschwankungen bei Patienten sind im Praxisalltag sehr häufig und konnten auch mit Studiendaten untermauert werden [2, 28]. Darüber hinaus konnten neuere Studien tageszeitliche Schwankungen der Achsenlänge sowie der untersuchten Strukturen im SD-OCT und auch in der OCT-Angiografie nachweisen [4, 18]. So wurden signifikante Korrelationen zwischen den täglichen Amplituden oder Akrophasen der choroidalen OCT-A-Indizes und der Aderhautdicke, dem intraokularen Druck und dem systemischen Blutdruck gefunden [18].
